# Applications of nanobodies in brain diseases

**DOI:** 10.3389/fimmu.2022.978513

**Published:** 2022-11-08

**Authors:** Fang Zheng, Yucheng Pang, Luyao Li, Yuxing Pang, Jiaxin Zhang, Xinyi Wang, Geert Raes

**Affiliations:** ^1^ The Key Laboratory of Environment and Genes Related to Disease of Ministry of Education, Health Science Center, Xi’an Jiaotong University, Xi’an, China; ^2^ School of Electronic Science and Engineering, University of Electronic Science and Technology of China, Chengdu, China; ^3^ Research Group of Cellular and Molecular Immunology, Vrije Universiteit Brussel, Brussels, Belgium; ^4^ Myeloid Cell Immunology Lab, Vlaams Instituut voor Biotechnologie (VIB) Center for Inflammation Research, Brussels, Belgium

**Keywords:** nanobody, brain disease, Alzheimer’s disease, Parkinson’s disease, brain infection, brain tumor, stroke, theragnostics

## Abstract

Nanobodies are antibody fragments derived from camelids, naturally endowed with properties like low molecular weight, high affinity and low immunogenicity, which contribute to their effective use as research tools, but also as diagnostic and therapeutic agents in a wide range of diseases, including brain diseases. Also, with the success of Caplacizumab, the first approved nanobody drug which was established as a first-in-class medication to treat acquired thrombotic thrombocytopenic purpura, nanobody-based therapy has received increasing attention. In the current review, we first briefly introduce the characterization and manufacturing of nanobodies. Then, we discuss the issue of crossing of the brain-blood-barrier (BBB) by nanobodies, making use of natural methods of BBB penetration, including passive diffusion, active efflux carriers (ATP-binding cassette transporters), carrier-mediated influx *via* solute carriers and transcytosis (including receptor-mediated transport, and adsorptive mediated transport) as well as various physical and chemical methods or even more complicated methods such as genetic methods *via* viral vectors to deliver nanobodies to the brain. Next, we give an extensive overview of research, diagnostic and therapeutic applications of nanobodies in brain-related diseases, with emphasis on Alzheimer’s disease, Parkinson’s disease, and brain tumors. Thanks to the advance of nanobody engineering and modification technologies, nanobodies can be linked to toxins or conjugated with radionuclides, photosensitizers and nanoparticles, according to different requirements. Finally, we provide several perspectives that may facilitate future studies and whereby the versatile nanobodies offer promising perspectives for advancing our knowledge about brain disorders, as well as hopefully yielding diagnostic and therapeutic solutions.

## 1 Introduction

### 1.1 Antibodies and nanobodies

**Figure 1:**
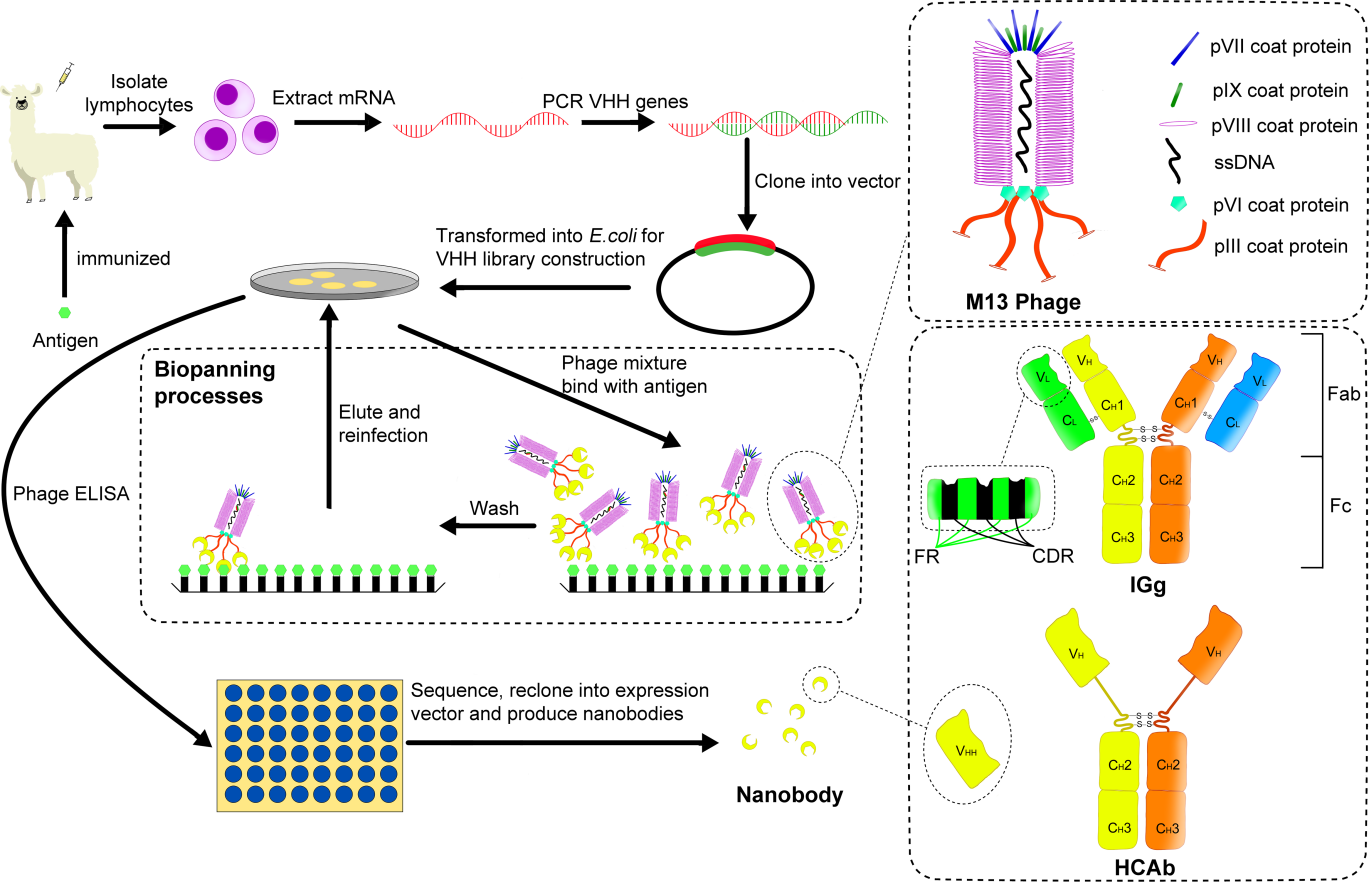
A brief process for building an immune library and screening for nanobody using M13 phage display. The coat structure of M13 phage is shown in the upper right panel, and the structure difference between general IgG and HCAb in camelids is shown in the lower right panel. After using a specific antigen to immunize a camelid, mRNA is extracted from its lymphocytes. Then PCR technology is used to obtain the corresponding DNA fragments, which are cloned into a phage display vector. After several cycles of biopanning, the required phages are screened for antigen binding via ELISA. Finally, the obtained nanobodies are sequenced and expressed for example in *E.coli*.

Antibodies form a basic Y-shaped structure consisting of two heavy chains and two light chains, of which different regions have different functions such as antigen-binding (Fab) and regulating the activity of immune cells (Fc), as shown in **Figure 1**. In addition, the half-life of IgG is prolonged by the binding and dissociation of the Fc part of IgG to the neonatal Fc receptor (FcRn) (1), and the affinity of the antibody to the antigen is most affected by the complementary determinant regions of the variable region.

In 1993, Hamers-Casterman et al. found a natural heavy chain antibody (HCAb) in camels containing only γ heavy chain and missing the first domain of its constant region (CH1) (**Figure 1**) (2). Subsequent studies have found that this HCAb is common in all camelids (3). The variable region of the HCAb that retains antigen-binding activity, namely the variable domain of heavy chain of heavy-chain antibody (VHH), has a molecular weight of only about 15 kDa (1/10 of that of conventional antibodies) and a size in the nanometer scale, while it retains an antigen binding affinity that is often in the low nanomolar range. Therefore, VHH was called nanobody (Nb) by its original developer, Belgian biopharmaceutical company Ablynx. Due to the small size of nanobodies (Nbs), they can be easily cleared by glomerular filtration and because they also lack an Fc-tail and the associated interaction with FcRn, their serum half-life is very short (4). The short serum half-life as compared to full size antibodies has major advantages for applications such as *in vivo* imaging, since it allows to obtain a rapid clearance from untargeted tissues, with a low background signal and high target-to-background ratio and thus high contrast within hours after tracer injection as compared to up to several days for full-size antibodies, and also limits the background radiation to untargeted organs and thus the radiotoxicity (5). On the other hand, as will also be discussed in section 2.2., protein engineering technology to combine multiple Nbs or make fusions with other proteins can be used to increase the Nb serum half-life for other applications, where a longer serum-half-life may be preferred, for example to obtain a sustained therapeutic effect. But this will then also again increase the size of these constructs and so it is necessary to make a trade-off between size and serum half-life, depending on the individual applications in research, diagnostics and therapy. It is worth mentioning that heavy chain antibodies have been found not only in camelids, but also in some cartilaginous fishes (such as nurse shark), named immunoglobulin new or nurse shark antigen receptor (IgNAR) to distinguish them from HCAb in camelids. In addition, their structures are different. For example, the variable domain of IgNAR (VNAR) only contains two CDRs, CDR1 and CDR3, and its CDR3 is usually longer than the corresponding region in IgG (6).

### 1.2 Screening and preparation of nanobodies

At present, the selection of Nbs is mainly realized by constructing a phage display library and screening high-performance antibodies from it.

For the construction of an immune library, the specific antigens (either as protein or *via* DNA vaccination) mixed with standard adjuvants such as Gerbu are injected into the camelid such as Bactrian camel, dromedary, llama and alpaca. These immunizations are performed typically eight times in two months and the corresponding HCAbs undergo affinity maturation (7, 8). Then an aliquot of peripheral blood is collected to obtain the lymphocytes and the mRNA in the lymphocytes is isolated and reverse transcribed into cDNA. Next, the cDNA can be amplified by PCR, cloned in frame to a surface protein gene on the phage vector, and then transferred into host cells such as *E. coli*. As a result, a small library containing the required VHH gene fragments (typically about 10^6^ individual transformants) is obtained (9). For reference, Pardon et al. provided comprehensive and detailed methods of immunization and the construction of immune libraries (10).

Phage display is a widely used display platform (11). The phages used include many types (T7, T4, M13, etc.), among which the filamentous phage of the M13 type is most commonly used. The M13 phage’s coat proteins include five types, as shown in **Figure 1**. Among all the coat proteins, the pIII protein is usually used as the display platform for Nb screening.

To select VHH’s binding the antigen with high affinity, progeny phages are typically subjected to two to three cycles of biopanning, whereby they are incubated on a microtitration plate which has antigen fixed on it. After washing of unwanted phages that are not immobilized on the antigen, the selected phages are eluted and used to re-infect the host. Finally, the individual clones are analyzed for antigen-binding by standard enzyme-linked immunosorbent assay (ELISA) and the corresponding genes are sequenced to complete the initial screening of Nbs (9). Among the identified Nbs, selection of the lead compounds is later on performed using functional assays, depending on the desired properties and applications. Since Nbs have no complex structure, they can be expressed in large quantities in hosts such as prokaryotic cells. The entire process of constructing an immune library and screening target antibodies by phage display is shown in **Figure 1**.

In addition to immune libraries, natural libraries (12) and synthetic/semi-synthetic libraries (13) have also been proposed for generation of Nbs. These libraries are not dependent on immune responses in experimental animals, but usually, the content of the libraries needs to be much higher than that of immune libraries, especially for natural libraries (~ 10^9^ individual clones) (3). In addition to phage display, the methods for screening the required Nbs from libraries also include cell surface display (14), ribosome display (15), mRNA/cDNA display (16), and high-throughput DNA sequencing and mass spectrometric identification (17), etc. There are also different suitable methods for different constructed libraries (9).

Nbs can be used as a good tool for research, diagnosis, and treatment. However, they need to be chemically functionalized to achieve certain functions in specific scenarios. Like for other proteins, chemical functionalization methods for Nbs can include natural amino acid residue methods, labeling methods, and non-natural amino acid methods (18). Using these methods, Nbs can be linked to fluorescent proteins (FP) or other detection moieties for imaging or to therapeutic moieties such as radionuclides, toxins or small molecule drugs as antibody-drug conjugates (ADC) for targeted therapy. In addition, Nbs can be formatted into a variety of other forms, such as the polyvalent Nb (polymer formed by the same Nbs) or multi-specific Nb (polymer formed by different Nbs). Compared with monovalent Nbs, these can have stronger apparent affinity or avidity for antigen (19, 20).

### 1.3 Brain-related applications of nanobodies as research tools

Thanks to their special structure, Nbs have many advantages compared to ordinary antibodies, such as high solubility (21, 22), high stability (23, 24), high antigen-binding ability (25), low immunogenicity (26), and strong tissue penetration (27). Based on these advantages, in addition to their clinical applications, which will be discussed later, Nbs can also be an excellent tool for basic brain research. In particular, to image the cell structure and reaction processes, Nbs tagged with fluorescent molecules are an excellent imaging tool in microscopy. Nbs were for example used to reveal an extra-synaptic population of synaptosomal-associated protein-25 (SNAP-25) and Syntaxin 1A in hippocampal neurons. Using a new technology called subdiffractional tracking of internalized molecules (sdTIM), based on a pulse-chase of fluorescently tagged ligands destined to undergo endocytic transport, the activity-dependent internalization of Atto647N-tagged anti-green fluorescent protein (GFP) Nbs bound to pHluorin-tagged synaptic protein vesicle-associated membrane protein 2 (VAMP2) was used to study the dynamics of endocytic pathways of synaptic vesicles (28). Similarly, antagonistic Nbs to vesicle glutamate transporters (VGLUTs) were linked to fluorescent molecules to study their inhibitory effect on neurotransmitter glutamate transport, improving the understanding of neurotransmitter transport processes (29). Nbs against activity-regulated cytoskeleton-associated (Arc) protein were used as a new tool for studying the dynamics and function of Arc protein, providing a new approach for studying the long-term plasticity, memory, and cognitive flexibility of synapses (30). In addition to small-scale imaging, a whole-body immunolabeling approach called Nb (VHH)-boosted 3D imaging of solvent-cleared organs (vDISCO) has been proposed. Hereby, the signal of endogenous fluorescent proteins expressed in the central nervous system of transgenic mice can be enhanced more than 100 times using Nbs targeting these fluorescent proteins that have in turn been tagged with potent dyes such as Atto594, Atto488 or Alexa647, allowing head-to-toe light slice microscopy (panoramic imaging) and subcellular detail imaging of transparent mice. This technology was used to image neuronal changes in different pathological conditions (31). To further improve the imaging quality of Nbs, in addition to fluorescent molecules, the combination of Nbs with quantum dots (QD) (32), single-walled carbon nanotubes (SWCNT) (33) and other materials also showed exciting results. SWCNT emit light in the near-infrared band without bleaching or scintillation, which is very advantageous for imaging deep tissues.

Nbs also have powerful functions as molecular linking tools, one of which is that the ribosomes in neurons retrogradely labeled with GFP can be targeted with anti-GFP Nbs, and this method allows immunoprecipitation of the mRNA being translated in the presence of GFP (34). For example, to study the dopamine pathways, researchers built a pseudorabies virus (PRV) strain, in which the spread of the virus and expression of GFP are activated only after exposure to cyclization recombination enzyme (Cre). Once activated in Cre-expressing neurons, the virus serially labels chains of presynaptic neurons. To further study edge dopamine neurons and their molecular characteristics of presynaptic input, researchers used the previously mentioned approach to immunoprecipitate ribosomes to retrograde trace infected neurons and identify important inputs to the mesolimbic dopamine pathway (35). In addition to the application in immunoprecipitation, Tang, J,CY et al. developed a method for gene manipulation using GFP and anti-GFP Nbs, called Cre recombinase dependent on GFP (CRE-DOG). In this set-up, GFP acts as a scaffold that aggregates modular transcription domains and assembles a hybrid transcription complex to activate the target gene, where GFP recognition is mediated by paired anti-GFP Nbs (36). Using similar ideas, the team also developed a GFP-dependent transcription system (37), termed flippase dependent on GFP (FLP-Dog) (38), and other gene manipulation methods, providing reliable tools for photogenetics and other technologies.

Of course, Nbs can also be used to study the role of certain molecules in diseases by taking advantage of antagonistic Nbs which can block the function of the targeted antigens. For example, von Willebrand factor (VWF) is an important factor affecting ischemic stroke. Through the construction of a targeted Nb, the specific role of von Willebrand factor in ischemic stroke could be studied and the mechanism of VWF mediated by its A1 domain could be determined (39). Finally, the Nb specificity can be used to identify specific molecules expressed in disease. For example, Jovcevska, I et al. enriched phage-displayed Nb libraries from protein extracts of glioblastoma cell lines NCH644 and NCH421K. Through bioinformatics analysis, several molecules, including dihydropyrimidinase-related protein 2 (DPYSL2), were identified that are expressed differentially in glioblastoma as compared to normal tissues, providing promising reference biomarkers for follow-up research and treatment (40).

## 2 Transport of nanobodies to the brain

Antibodies usually perform their functions by binding to target molecules, which means that we need to deliver Nbs to the brain in order to have functionality in the brain. The blood-brain-barrier (BBB) is the biggest obstruction to drug delivery in the brain, and whether the BBB is crossed naturally by the Nb or not determines how the drug needs to be administered. In general, administration methods which themselves cross the BBB, such as intrathecal injection, can usually result in a higher concentration of drugs in the brain, but have the risk of greater tissue injury. On the other hand, drug delivery across the BBB after intravenous injection, is characterized by low tissue damage, but usually also lower concentration of drugs in the target organ. Both methods have their advantages and disadvantages. Nowadays, minimally invasive or even non-invasive diagnosis and treatment methods have received most attention. How to reduce the damage of drug administration and make it pass the BBB efficiently is one of the focuses of research. Compared with ordinary antibodies, Nbs have a 10 times smaller size and thus they should at least in theory have a better chance of penetrating the BBB, which may offer advantages for non-invasive or minimally invasive treatment.

### 2.1 Natural methods of BBB penetration

**Figure 2:**
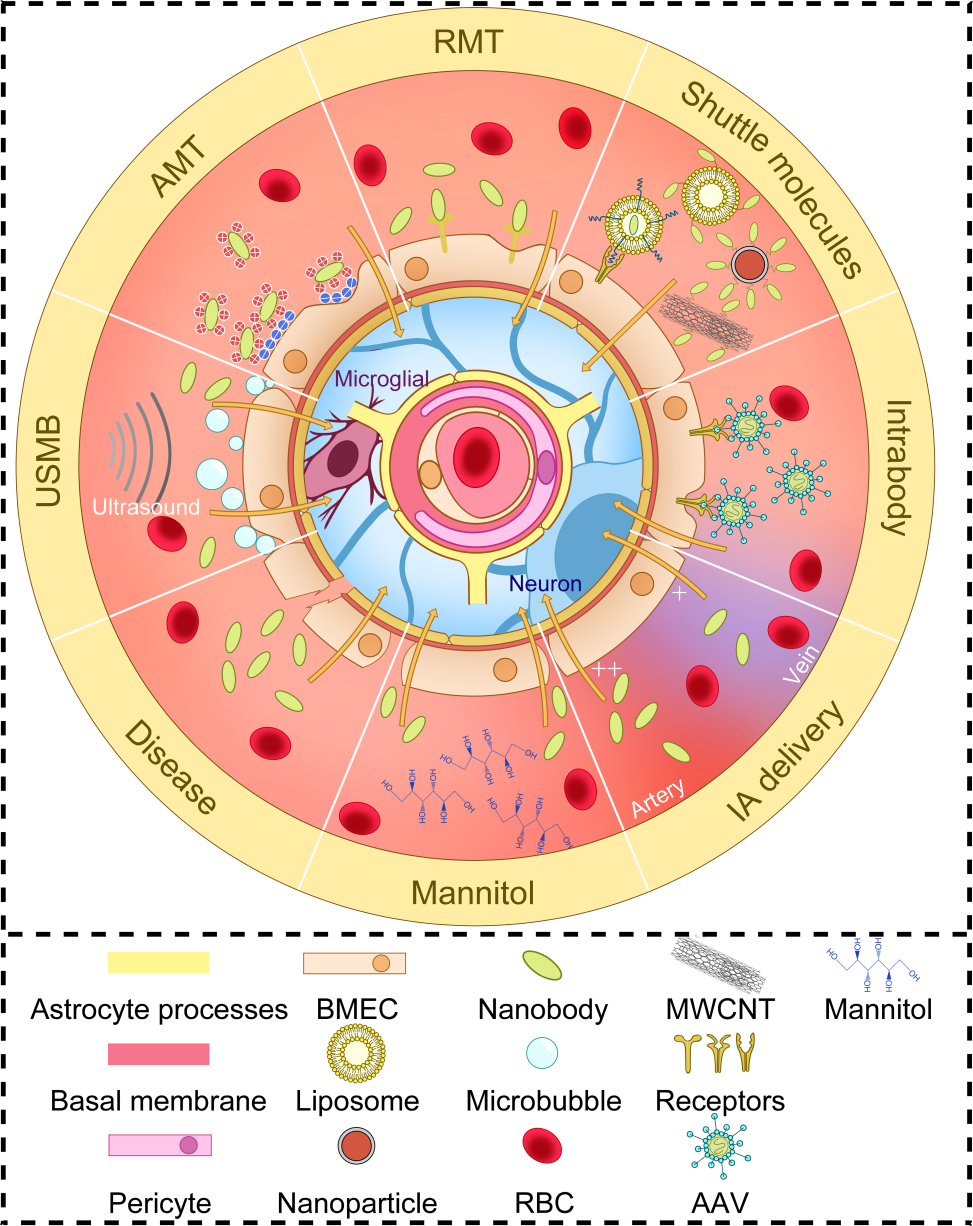
The structure of neurovascular units and several ways to increase BBB delivery of nanobodies. Brain-microvessel endothelial cells (BMECs), astrocytes, pericytes, capillary basal membrane, microglia, and neurons make up neurovascular Units (NVU). The efficiency of nanobodies crossing the BBB depends on altering BBB permeability or some physiological mechanisms of BBB crossing. AMT, adsorptive-mediated transcytosis; RMT, receptor-mediated transcytosis; USMB, ultrasound and microbubble; MWCNT, multi-walled carbon nanotubes; AAV, adeno-associated virus vectors; RBC, red blood cell; IA, intra-arterial.

Barrier structures in the human body protect import organs, such as the blood-testosterone barrier, blood-eye barrier, blood-placenta barrier, etc. Among them, the BBB, which protects the central nervous system (CNS), is the barrier structure that is most difficult to pass in the human body. The average microvascular surface area per gram of tissue is 150 to 200 square centimeters, and the average BBB area of an adult is 12 to 18 square meters. The production and maintenance of BBB function mainly depends on the interaction between brain-microvessel endothelial cells (BMECs), and astrocytes and pericytes. These structures, capillary basal membrane, microglia, and neurons are collectively called neurovascular units (NVU) (as shown in **Figure 2**) (27, 41). In physiology, the BBB allows molecules to pass through in several ways. The first way is passive diffusion. Fat solubility and molecular weight determine the efficiency of passive diffusion. When the molecular weight of a molecule is greater than 400Da, fat solubility does not increase the efficiency of its penetration through the BBB. At the same time, a high polar surface area (PSA) greater than 80 Å2 and the tendency to form more than 6 hydrogen bonds are also considered limiting factors for the entry of compounds into the central nervous system. The second pathway is active efflux. ATP-binding cassette proteins (ABC proteins) are expressed in the endocortical membrane of the blood-brain barrier. They lead to drug resistance characteristics of the CNS through the active efflux of foreign molecules and endogenous metabolites. The third pathway is carrier-mediated transport (CMT) through specific carrier proteins. Amino acids, fatty acids, and other substances cross through the BBB in this way. The fourth pathway is transcytosis, which includes receptor-mediated transcytosis (RMT), whereby larger molecules initiate transcytosis either by binding to specific receptors, and adsoptive-mediated transcytosis (AMT), which is based on adsorption with a positive charge to specific sites in the cell membrane (41).

### 2.2 BBB penetration by nanobodies after systemic administration *via* blood

Nbs use similar physiological mechanisms to pass through the BBB as those described above (**Figure 2**). Thereby it should be taken into account that the concentration of the Nb that can be obtained in the brain, depends not only on the ability of the Nb to cross the BBB but also on its pharmacokinetic characteristics. Studies using multivalent constructs of Nbs to increase their avidity or using Nb constructs of which the serum half-life had been increased *via* PEGylation or fusion with another Nb targeting serum albumin, have revealed that affinity and circulation time affect the uptake of Nbs in the brain (20, 42). In addition, the fusion of Nbs with an Fc segment can also increase circulating plasma half-life and increase brain uptake (43). It is important, however, to note that fusing Fc segments and Nbs alone may not necessarily be sufficient to increase brain uptake, since in another study fusing Fc segments to extend the time window for brain uptake did not increase BBB crossing, which may be related to molecular weight, species differences, or whether the Nbs themselves penetrate the BBB (44).

A first method *via* which some Nbs can cross the BBB is *via* RMT. The first Nbs described to cross the BBB *via* RMT were FC5 and FC44 (45). FC5 binds to BMECs’ luminal α (2, 3)-sialoglycoprotein receptor to trigger clathrin-mediated endocytosis (46), FC44 binds to a protein of about 36kDa on BMECs, of which the identity needs further study (45). Compared with Nbs EG2 and A20.1 (targeting extracellular domain of EGFRvIII and *C. difficile* toxin A, respectively), FC5 and FC44 passed through an *in vitro* BBB model (SV-ARBEC) more easily (50-100 folds) and also had a better CSF/serum concentration ratio (20-40 folds) *in vivo (* 47). Fusing the FC5 Nb to a human Fc segment, improved serum pharmacokinetics and resulted in an enhanced pharmacological action of chemically coupled agents. A bivalent fusion of FC5 with Fc exhibited a significantly increased transcytosis rate across the cerebral epithelial monolayer as compared to a monovalent fusion (43). Nbs targeting insulin-like growth factor receptors have been studied as tools for brain delivery of biologics. In one study, three Nbs targeting the extracellular domain of the human insulin-like growth factor-1 receptor (IGF1R) showed transmigration across an *in vitro* cell line-BBB model (SV-ARBEC). *In vivo*, fusions of these Nbs with mouse Fc segment showed enhanced brain uptake *via* RMT as compared to a control mFc. One of the IGF1R Nb-mFc coupled with the non-BBB crossing analgesic neuropeptide galanin also showed a dose-dependent analgesic effect (48). More recently, Nbs targeting the transferrin receptor have been produced as tools to deliver a biologically active peptide to the brain *via* RMT. Peripheral injection of such Nbs coupled with neurotensin, a neuropeptide that causes hypothermia when present in the brain but is unable to reach the brain from the periphery, resulted in hypothermia in mice (49). Finally, also an anti-prion Nb, known as PrioV3, was reported to be capable of crossing the BBB *in vitro* and *in vivo via* RMT (50).

A limited number of Nbs have been reported to pass through the BBB spontaneously *via* AMT. These Nbs generally have higher pI (27), such as a Nb targeting GFAP (pI=9.4) (51), R3QV targeting Aβ42 (pI>8.3) and A2 targeting Phosphorylated Tau protein (pI>9.5) (19).

A third approach is to deliver Nbs across the BBB using structures that can cross the BBB, such as glutathione targeted PEGylated liposomes (52), multi-walled carbon nanotubes (MWCNTs) (53), or Fe_3_O_4_ nanoparticles (54). On the other hand, Nbs can themselves be used to improve the targeting of liposomes in the brain (55). It is worth noting that cell penetrating peptides (CPPs), may also be useful for increasing Nb penetration into the brain. Fusing Nbs to CPPs has allowed the generation of cell-permeable Nbs, that can enter into living cells *via* non-endocytic delivery (56). Although the internalization process of this short amino acid sequence with positive charge is still controversial, it has shown the ability to help scFv targeting PrP^Sc^ and α-synuclein penetrating the BBB, which should also be applicable to Nbs (57, 58).

In addition, a temporary increase in BBB permeability through physical and chemical means can also improve the ability of Nbs to pass through the BBB. Studies have shown that intra-arterial administration can increase the uptake of Nbs in the brain (59). Mannitol, as a hyperosmotic agent, can also improve the ability of several Nb fusion molecules to cross the BBB by weakening the junctions of BMECs (48, 51, 59). In a recent study, the combination of ultrasound and microbubbles (USMB) was found to enhance the permeability of drugs through an *in vitro* epithelial cell (MDCK II) barrier. USMB treatment increased epithelial cell permeability to large molecular weight molecules (between 4 and 20 kDa) by 3-7 times while promoting the intracellular accumulation of the same molecules. USMB doubled the paracellular permeability of an anti-CXCR4 Nb and increased its binding to retinoblastoma cells five-folds, suggesting a possible novel way to improve the delivery of drugs to organs protected by tissue barriers such as the BBB (60). The increase in brain temperature is also one of the factors affecting the structural stability of the BBB (27). Finally, when the BBB is damaged by disease, the ability of Nbs to pass through the BBB is naturally enhanced. This is why many studies on disease-related Nbs show that they can spontaneously enter the brain under pathological conditions where the BBB integrity is compromised (51, 61).

### 2.3 Other administration methods of nanobodies used for brain targeting

Less commonly used administration methods for Nbs include intranasal, intrathecal/intraventricular administration, and other local administration methods. For example, intracerebroventricular injection of Aβ -secretase 1(BACE1)-inhibiting Nb was reported to induce acute reduction of Aβ load in blood and brain of transgenic AD model mice (62). Intrathecal administration, which induces less damage than intraventricular injection and has been extensively studied in stroke and neurodegenerative disease models to deliver larger molecules across the BBB (63), may also be an interesting approach to deliver Nbs in the brain in the future. Pizzo et al. have analyzed how IgG and Nbs distribute throughout the rat brain in a size-dependent manner after intrathecal administration. Both spread in the brain surface, and along the perivascular space around all types of blood vessels studied, whereby the percentage brain area accessed was strikingly greater for the smaller sdAb than for the larger IgG (64).

Drugs delivered by intranasal administration can cross the BBB by intracellular or paracellular pathways of the olfactory nerve and trigeminal nerve (65), which is a good method of local administration for drugs that cannot cross the BBB. Notably, intranasally administered Nb against Transthyretin (TTR) was found to cross the BBB. However, in that case also target-mediated effects occurred, whereby in wild-type mice, the anti-TTR Nb was specifically internalized in a receptor-mediated process by motor neurons, whereas in TTR deficient mice, the Nb was internalized by all cells, for late lysosomal degradation (66).

In a completely different approach from the protein-based methods, gene transfer strategies with adeno-associated virus (AAV) vectors can deliver Nbs directly to the CNS in a manner that rather transports genes, and thus has long-term effects (67).

## 3 Diagnostic and therapeutic applications of nanobodies in brain diseases

### 3.1 Alzheimer’s disease

Alzheimer’s disease (AD) is a degenerative disease of the CNS. In addition to macroscopic manifestations of brain atrophy, pathological phenomena in AD patients’ brain tissues include tau-containing neurofibrillary tangles, amyloid β-protein (Aβ) plaques, activated glia or enlarged endosomes, which can be observed by microscopy. Also covert changes such as loss of synaptic homeostasis, loss of integrity of neurons or neuronal networks occur (68). The current diagnosis and treatment of AD are based on biomarkers related to its pathology and clinical manifestations of cognitive impairment. Although the etiology of AD is unknown, symptoms of cognitive impairment in AD can be detected by neuropsychological testing. However, multiple brain diseases may cause similar symptoms, so using biomarkers to achieve differential diagnosis and clinical staging is a solution. The biomarker diagnosis of AD is summarized as the ATN framework, i.e., “Aβ”, “Tau” and “neurodegeneration”, including A: Aβ-PET (Positron Emission Computed Tomography) of the brain, Aβ42 in body fluids or Aβ40/Aβ42, T: Tau-PET, P-Tau181 or P-Tau217, N: FDG(Fluorodeoxyglucose)-PET or MRI (showing brain atrophy and distinguishing it from other diseases such as cerebral hemorrhage), Total Tau, Neurofilament light (NfL), etc. (68). Aβ, P-Tau, and NfL in body fluids can be simply detected by ELISA. The tracers used for Aβ-PET are ^11^C Pittsburgh Compound-B (PIB), ^18^F-Flutemetamol, ^18^F-Florbetapir and ^18^F-Forbetaben. The tracers used for Tau-PET is ^18^F-Flortaucipir (68–70). Although antibodies are widely used in ELISA and other technologies, antibodies have at present not been used in the imaging diagnosis of AD, and no image-related antibody tracer has entered clinical trials. Some preclinical imaging studies have started to report the use of Nbs for detecting AD-related biomarkers. For example, despite a brain uptake yet too low for effective *in vivo* imaging, ^99m^Tc-labeled Nb pa2H targeting Aβ resulted in a small yet significant higher cerebral uptake in APP/PS1 mice (71). R3QV, a Nb binding the central 17~28 residues of Aβ, and Nb A2 targeting the C-terminal of pTau protein, both have been reported to cross the BBB (19). Site-specific labeling of R3QV with the contrastophore gadolinium was used to design imaging probes for magnetic resonance imaging (MRI) (72). In addition, gadolinium-based nanoparticles were also chelated with radionuclies (^68^Ga^3+^ or ^111^In^3+^) or covalently bound with near-infrared pigments (such as Cyanine5.5) to be used *in vivo (* 73).

Notably, Nbs targeting Aβ oligomers (PrioAD12 and PrioAD13) detected accumulation of Aβ oligomers in the retina, which originated in peripheral blood and preceded cognitive decline and brain deposition of oligomers. This provides an idea for early detection of AD *via* an “eye test” (74, 75). In addition to the identification of typical pathological molecules, the identification of different forms of aggregation of the Aβ molecule is also of interest because they appear in AD at different stages of the disease. The V31-1 Nb recognizes the C-terminal of Aβ42 and only recognizes monomers and oligomers in Western Blot (WB) and dot-blot analysis (76). Similarly, Nbs A4 and E1 exhibit strong affinity for Aβ oligomers (77, 78). Another Nb, B10, showed recognition of the Aβ fibril without recognition of the protein fragment Aβ1-40 or weakly of the Aβ oligomers, indicating recognition of conformational epitopes (79). In addition, Rutgers et al. constructed eight Nbs targeting Aβ (ni3A, ni8B, va2E, vaE2, va1G, pa4D, pa11E, pa2H), among which pa4D, pa11E, and pa2H can recognize Aβ in the vascular and parenchymal depositions, while the other Nbs can only recognize Aβ in the vascular depositions. This suggests that there may be differences between Aβ epitopes in blood vessels and brain (80). Among various tested Aβ-targeting Nbs, VHH ni3A showed the highest transmigration efficiency in an *in vitro* blood-brain barrier model (co-culture of bovine brain capillary endothelial cells and rat’s astrocytes) through active transport (81). Tau protein is also one of the targets for AD, for example using Nb F8-2 targeting the C-terminal of Tau protein (82).

Currently, the treatment methods of AD are not very effective. In addition to conventional treatment of complications and improvement of living habits, cholinesterase inhibitors such as Donepezil and Rivastamine and similar drugs can only improve the functional abnormalities caused by cholinergic neuron damage in AD patients. Disease-modifying therapies targeting AD pathological molecules have been widely studied, and many antibody drugs have entered clinical trials (68, 83). On 2 November 2019, the State Medical Products Administration of the People’s Republic of China (SDA) conditionally approved Oligomannate, an acid oligosaccharide extracted from Marine brown algae, for marketing with the pharmacological action of preventing Aβ fibrils from forming and depolymerizing the formed fibrils. The U.S. Food and Drug Administration (FDA) announced On June 7, 2021 that it has approved Biogen’s Aducanumab for the treatment of early-stage Alzheimer’s disease (AD). Although its therapeutic efficacy is still debated, there is no doubt that it has led to the development of AD antibody drugs and disease-modifying therapies.

The use of Nbs to treat AD has also been investigated. Nbs can play a therapeutic role by inhibiting fibrils aggregation and blocking Aβ -induced neurotoxicity. Aβ oligomers were found to be primarily toxic molecules rather than mature Aβ fibrils or Aβ monomers. Nbs A4, E1, V31-1 can bind to Aβ oligomers, inhibit their aggregation into Aβ fibrils and block Aβ oligomer induced neurotoxicity to SH-SY5Y neuroblastoma cells (76–78). Similarly, B10AP prevents the formation of mature Aβ amyloid fibrils by preventing the formation of fibrils in the protofibrils stage (the stage before the formation of mature Aβ fibrils) (79). In addition to inhibiting fiber aggregation, direct degradation of Aβ is also a feasible method. Asec-1A could inhibit the toxicity of Aβ aggregation to SH-SY5Y neuroblastoma cells by hydrolyzing Aβ at the α-secretase site (84, 85). In addition, the fusion of Nbs that can cross the BBB with therapeutic molecules was also evaluated. In a longitudinal study with treatment for 5 weeks using a BBB crossing Nb (FC5) fused to an amyloid-β oligomer-binding peptide (ABP) *via* a mouse IgG Fc fragment, PET showed a significant reduction in brain Aβ levels, MRI showed correction of hippocampal atrophy and increased CSF Aβ ratio of 42/40 and decreased Nfl concentration was found (86). Although Aβ is the primary target for the treatment of AD, improvement in Tau pathology is also of therapeutic significance. VHH Z70 recognizes the PHF6 sequence, known for its nucleation capacity, and effectively inhibits Tau aggregation *in vitro* and spread and aggregation in FRET biosensor reporter cells. Local expression of VHH Z70 in the brain using a lentiviral vector infection also reduced tau spread in the Tau pathological mouse model THY-Tau 30 (87). As a method of treatment, AAV-mediated delivery of Nbs into the brain has also been evaluated, namely for the Nb targeting BACE1 (beta-secretase 1), a target in Alzheimer’s disease (67). Nbs related to the diagnosis and treatment of AD are summarized in **Supplementary Table 1**.

### 3.2 Parkinson’s disease

Parkinson’s disease (PD) is a neurodegenerative disease of which the incidence increases with age. Its etiology is complex and is explained by gene environment interactions. The current COVID-19 pandemic has raised concerns that COVID-19 may increase the risk of PD sickness, because COVID-19 leads to low body temperature and may cause neural degeneration following nasal entry into the brain (88). Currently it is believed that the pathophysiological characteristics of PD are the accelerated death of dopaminergic neurons caused by the complex interaction of abnormal α-synuclein aggregation with lewy bodies, mitochondrial dysfunction, lysosomes or vesicle transport, synaptic transport problems and neuroinflammation. Macroscopic manifestations are symptoms caused by the loss of dopamine cells in the substantia nigra striatum, such as typical bradykinesia and less common depression and anxiety, etc. The symptoms of disease are usually treated by levodopa (a precursor of dopamine) (88).

At present, the diagnosis of PD mainly depends on symptoms, that is, it does not meet the absolute exclusion criteria, there are at least two supportive criteria, and there are no red flags (89). However, diagnosis or scale dependent on clinical manifestations cannot avoid some subjectivity, and diagnosis combined with biomarkers will improve this shortcoming and be more conducive to early diagnosis.

Nbs developed so far in the context of PD mainly target α-synuclein, including NbSyn2 and NbSyn87 (90–93), which both target the C terminal of α-synuclein, but the sequence targeted by NbSyn2 is closer to the C terminal, and has less impact on the aggregation of α-synuclein (93). Studies have shown that selective phosphorylation of α-synuclein will significantly change the affinity of NbSyn87 binding to it (94). Recently, Nbα-syn01, a Nb targeting the n-terminal of α-synuclein, has also emerged, and preferentially recognizes α-synuclein fibrils rather than monomers (95). However, as far as PD diagnosis is concerned, so far there have not been any Nb studies related to brain imaging of diagnostic biomarkers.

Concerning medical treatment for PD, the ideas for reducing the pathological aggregation of α-synuclein can be divided into two ways: the first is to inhibit the aggregation of α-synuclein into fibrils, and the second is to degrade α-synuclein. Studies have shown that both NbSyn2 and NbSyn87 can inhibit the formation of α-synuclein fibrils *in vitro*, and their presence reduces high forster resonance energy transfer (FRET) α-synuclein oligomer with greater toxicity, which in turn reduces cell death, microglial activation and other injury responses (96). Nba-syn01 can also inhibit the α-synuclein-seeded aggregation *in vitro* and reduce its toxicity to SH-SY5Y cells (95). Since α-synuclein in PD is mainly aggregated inside cells, its degradation becomes more difficult. Constructing Nbs as intrabodies will allow them to be expressed in cells. Meanwhile, fusion of intrabodies with PEST amino acid sequences rich in prolyl(P), glutamine(E), aspartic acid(D), serine(S) and threonyl(T) residues can improve the intracellular solubility of intrabodies and induce proteasome degradation (97). As such, NbSyn87PEST intrabodies could degrade α-synuclein aggregation in cell culture and reduce its cytotoxicity (98). In subsequent studies, adeno-associated virus vectors were injected into the substantia nigra through stereotaxic injections and it was found that NbSyn87PEST intrabodies could significantly reduce the pathological aggregation of α-synuclein. However, compared with human sourced scFv VH14, NbSyn87PEST showed no significant dopaminergic tone maintenance in the striatum, and there was a certain inflammatory response (99).

In addition to targeting α-synuclein, targeting other PD-related proteins is also a treatment method. For example, since PD is usually associated with mutations of Leucine-rich repeat kinase 2 (LRRK2) that increase its activity, a variety of Nbs targeting different sites of LRRK2 have been developed as inhibitors (100, 101). Nbs associated with PD diagnosis and treatment are summarized in **Supplementary Table 2**.

Recent studies have found that the pathology of PD occurs in blood, skin and other tissues. Although the specificity and sensitivity of its diagnosis using peripheral markers are not all high, this provides ideas for the development of diagnosis in the future (102). In addition to the typical PD-related molecules, damage-related molecules common to some diseases can also be used as therapeutic or diagnostic targets, such as Nbs targeting caspase-3 and Mitochondrial Rho GTPase 1 (Miro1). Nbs against the former can resist cell damage caused by oxidative stress in a variety of brain diseases, while the latter is associated with mitochondrial homeostasis and is a potential biomarker or therapeutic target after visualization or regulation by Nbs (103, 104).

### 3.3 Brain tumor

#### 3.3.1 Glioblastoma

Isocitrate dehydrogenase (IDH)-wildtype glioblastoma (GB), previously termed as primary glioblastoma, is the most common and malignant glioma occurring in adults (105), with a peak incidence in the age group of 75 to 79 years old (106), especially among Caucasians (107). Current standard treatment for GB upon initial diagnosis is surgical resection followed by the postoperative Stupp protocol, namely radiation and concomitant chemotherapy with temozolomide (108). Although the application of the Stupp protocol since 2005 has significantly enhanced therapeutic effects, the median survival of patients still doesn’t exceed two years (109), for which the essential reasons lie in the nature of the tumor. This WHO grade IV diffuse glioma is characterized by heightened invasiveness, intra- and inter-tumor heterogeneity, relative resistance to therapy, and high recurrence rate, mainly due to the mobility of glioblastoma stem cells (GSCs). It has a profoundly immunosuppressive microenvironment mediated by myeloid-derived suppressor cells (MDSCs), and further affects the systemic immune status even without distant metastases. Furthermore, gliomas exhibit a low mutational burden and offer few therapeutic targets to the immune system, severely limiting the efficacy of immunotherapy (110). Given the lack of visual signals of all the tumor cells, part of the cancer tissues remain inevitably after surgery. Meanwhile, functional areas of the brain are frequently involved, which poses challenges for surgeons to achieve the balance between maximizing the extent of resection and minimizing neurological morbidity. Further, the BBB remains a formidable hurdle for drug delivery.

Although the molecular parameters of glioma have become important criteria for GB classification and grading (105), current clinical diagnosis still mainly relies on imaging examination including CT, MRI and PET/SPECT. However, CT/MRI can only observe the anatomical features of the tumor, and the relatively high uptake of glucose by the cerebral cortex makes the imaging specificity of 18F-FDG for brain tumors not high (111). Not only this, the full course of glioma treatment requires highly sensitive, non-invasive imaging strategies to demarcate tumor boundaries, guide surgery, evaluate therapeutic effects, and identify recurrence.

Given the difficulties in the diagnosis and treatment of GBs, Nbs are attracting attention. A dual-target Nb aiming at EGFR/EGFRvIII (wild type and mutant EGFR) -overexpressed in GB cells, was labelled with Cy5.5 (a near infrared dye) for optical imaging (112). Further, the Cy5.5-labelled Nb was attached to small unilamellar vesicles loaded with the MRI contrast agent Gd, achieving MRI/NIR bimodal imaging (55). However, considering the heterogeneity of GB, researchers have tried to find target binding antigens in the tumor microenvironment, including the overexpressed insulin-like growth factor binding protein 7 (IGFBP7) in the blood vessels of brain tumors (54), as well as signal regulatory protein alpha (SIRPα) significantly expressed by microglia- and monocyte-derived tumor-associated macrophages (TAMs) (113). A recent study reported that anti-SIRPα Nb labeled with ^99m^Tc can qualitatively display intracranial tumors (113).

A basic idea of applying Nbs for GB treatment is to antagonize key antigens in the progression of glioma. Zottel A et al. first validated eight novel antigens of GB and then examined the effects of four Nbs and their different combinations on glioma cells *in vitro*. Among them, Nb225 (anti-TUFM) and Nb79 (anti-vimentin) showed good tumor suppressive effect, with no harm to normal astrocytes (114). As an alternative, Nbs have been used as the targeting moiety of immunotoxins, setting the stage for the cytotoxic part to come into play. Some studies have used bivalent anti-EGFR Nb coupled with pseudomonas exotoxin (PE) to potentiate killing of GB tumor cells and tumor stem cells by pro−apoptotic tumor necrosis factor−related apoptosis−inducing ligand (TRAIL). Hereby, the receptor-targeted toxin upregulated TRAIL death receptors and suppressed the expression of anti−apoptotic proteins, thus overcoming TRAIL resistance in BG tumor cells and tumor stem cells (115, 116). It is worth noting that a recent study by Porčnik A et al. focused on the molecular signatures and driver genes behind the cellular subpopulations of GB, and managed to relate tumor invasion with the expression pattern of the transcription factor TRIM28 in the core and rim of the tumor (117). Based on these findings, they generated Nb237 to antagonize TRIM28 for reducing the invasiveness of GB cells, and obtained favorable results *in vivo* using zebra fish embryos xenografted with GB cells.

In recent years, photoimmunotherapy (PIT) has achieved great progress. It combines near-infrared light, which is harmless to the human body, and immunotherapy to selectively kill cancer cells overexpressing specific antigens through the activation of the light-activated substance IRDye700DX. Researchers are also trying to apply this new strategy in the treatment of GB. It was reported that US28, a foreign viral GPCR encoded by human cytomegalovirus and expressed by tumor cells, enhanced GB growth (118). Thus, a US28-targeting bivalent Nb was developed, and further labelled with IRDye700DX for PIT (119). An EGFR antibody-photosensitizer conjugate AkaluxTM has been commercially approved for head and neck malignancies (NCT02422979). Using Nbs as targeting substances, PIT may open a new chapter in glioma treatment.

#### 3.3.2 Brain metastases

Brain metastases (BMs), the major type of adult brain tumor, occur when primary tumor cells migrate through blood, lymphatic or directly invade the brain. The most common primary tumors giving rise to BMs are lung cancer, breast cancer and melanoma, with proportions of 40%-50%, 15%-20%, and 5%-20% respectively (120). With the progress of radiotherapy and molecular targeted therapy, consensus has been reached, that an individualized treatment plan should be developed for patients with BM, using the optimal sequential use of surgery, stereotactic radiosurgery, whole-brain radiation therapy, targeted therapy, and immunotherapy (121). However, given the difficulty of treatment, prevention of BMs should be the focus, especially among the high-risk groups, including patients with stage III/IV non-small cell lung cancer (NSCLC), HER2+ breast cancer, and melanoma.

As compared to studies on Nbs for the theragnostics of GB, research on the use of Nbs in the context of BMs is relatively mature, probably because people have a better understanding of the pathological mechanism of the primary tumors and can continue to use their well-developed Nbs. A notable example is the attempt to utilize 2Rs15d for detecting and treating the BMs of HER2pos breast cancer. 2Rs15d binds to the domain I of HER2, different from the epitope recognized by trastuzumab and pertuzumab (C-terminus of domain IV) (122) and maintains its affinity to HER2 under conditions where the individual is receiving HER2-targeted drug therapy, which enables 2Rs15d derivatives to be used either as imaging probes for monitoring the treatment effect or serve as an add-on treatment. In an early study, 18F labeled 2Rs15d injected intravenously into mice bearing intracranial HER2-expressing BT474M1 tumors, successfully visualized the HER2pos brain tumor *via* PET, with tumor-to-tissue ratios greater than 10:1 in major organs except for kidney (123). When radiolabeled with 111In, similar results were also observed in mice bearing either intracranial SKOV3.IP1 or 231Br tumors *via* μSPECT/CT (124). Quantum dots (QDs), also known as semiconductor nanocrystals (semi-conductor nanocrystals), have unique optical properties compared with traditional dyes, and can detect target molecules with extremely low concentrations, thereby significantly improving detection sensitivity. According to one study, anti-HER2 Nb fluorescently labelled with QDs could efficiently assess micro-metastases even in thick tissue sections using single- and two-photon imaging (125). Besides, in this study, breast cancer cells were first transplanted into the peripheral organs and tissues of animals to better simulate the process of BMs. Overall, these results demonstrated the potential of Nb-based imaging agents in BMs detection.

Further, radiolabeled 2Rs15d has exhibited therapeutic potential. The same study where 111In-2Rs15d was introduced, also coupled 2Rs15d with α−particle emitting 225Ac or β−particle emitting 131I (31). Mice bearing intracranial tumors receiving either 225Ac-2Rs15d or 131I -2Rs15d both had a longer median survival. Thereby, an α-radionuclide such as 225Ac, an excellent therapeutic candidate, may expand treatment options in addition to β−particle radiotherapy. It is worth noting that 131I can release β- particles and γ-rays simultaneously, showing theragnostic value.

Moreover, taking advantage of Nbs, efforts have been made to tackle BMs of NSCLC. Precision medicine is the most distinctive feature of lung cancer diagnosis and treatment. Targeted therapy has become the core method for the treatment of advanced (NSCLC), especially for relatively well-researched targets such as EGFR and ALK. However, this also faces a serious problem: resistance mutations of tumors. For example, EGFRT790M is the major cause of the failure of first-generation EGFR TKI, represented by gefitinib. In addition, when tumors metastasize to the brain, drugs that do not easily penetrate the BBB also lose their usefulness. To address the core challenges of NSCLC BM and drug resistance, Yin W et al. constructed a sophisticated liposomal system, T12/P-Lipo (126), in which they attached a transferrin receptor (TfR)-binding peptide T12 and an anti-PDL1 Nb to the surface of the liposome. PD-1/PD-L1 monoclonal antibody is a representative immune checkpoint inhibitor (ICI). Although PD-L1 is not upregulated in primary brain tumors, it is highly expressed in NSCLC cancer cells, the surrounding TAMs and tumor vascular epithelial cells. Hence, anti-PDL1 Nbs can be effectively used as targeting ligands for nanocarriers. Regarding the latter, simvastatin (SV) and gefitinib (Gef) were loaded into the engineered liposome, whereby SV could promote the repolarization of TAM from M2 to M1, thereby re-sensitizing EGFRT790M cells to Gef. The therapeutic efficacy of T12/P-Lipo was thus evidenced in the BMs model of H1975 NSCLC(**Supplementary Table 3**).

### 3.4 Other brain diseases

Antibodies or antibody fragments such as Nbs are rarely used in infectious diseases of the central nervous system. In a recent study, Nbs VHHG9 and VHHF3 targeting Neisseria adhesin A (NadA), which can antagonize the interaction between recombinant NadA and cell receptors, were developed. The preincubation of Neisseria meningitidis with VHHF3 and VHHG9 could significantly reduce the adhesion of *neisseria meningitidis* to human microvascular endothelial cells *in situ* and prevent it from crossing a BBB model (human BMECs) *in vitro*, providing a new treatment idea (127). Rabies is also an infectious disease in which the CNS is mainly affected. After infection, the virus invades the CNS through the peripheral nervous system, presenting symptoms such as hydrophobia and mania. There is a high risk of death from respiratory or circulatory failure without early treatment, so post-exposure treatment of rabies is very important. The use of antibodies to neutralize the virus is a very effective way to reduce the cost of post-exposure treatment. A variety of Nbs with high affinity and low cost have been developed. For example, Nbs were linked with a coil-coil peptide derived from the human cartilage oligomeric matrix protein (COMP48) to form homogenous pentavalent multimers called combodies 26424 and 26434] (128) or bi-specific Nbs were developed that can target albumin to increase blood half-life, which were then combined with post-exposure vaccine prophylaxis (129, 130).

Prion diseases are rare CNS degenerative diseases of specific etiology. They can be classified as sporadic, genetic, or acquired (infection), such as Creutzfeldt-Jakob disease, fatal familial insomnia, and Kuru disease. They are characterized by accumulation and aggregation of prions or abnormally folded proteins. Compared with the typical α-helix in normal PrP^c^ (Cellular Prion protein), the abnormally folded isoform PrP^sc^ has a large number of β-folds and is partially resistant to proteases, rapidly converting normal proteins into abnormal proteins (131). Due to the special pathological manifestations and specific pathological molecules of the disease, the diagnosis is relatively clear (such as brain MRI and immunohistochemical detection of PrP^sc^ deposition, etc.), but there is no clear treatment plan. At present, research on the use of Nbs in the field of prion diseases has mainly been focused on studying of the molecular interaction and developing a potential treatment. For example, NB-PRP-01 targeting PrP^c^ was reported to be co-crystallized with PrP^c^ (132). The mechanism of PrP transformation was studied by the crystallization of Nb484 and full-length human PrP (133). It was further found that Nb484 could bind to the hydrophobic region of mouse PrP, inhibit its transformation and showed no neurotoxicity in cultured sections (134). Nbs that simultaneously could cross the BBB and have therapeutic effects will have the best therapeutic significance. For example, PrioV3 Antibody has been found to traverse *in vitro* or *in vivo* the BBB and inhibit PrP^SC^ accumulation in ScN2a Cells and ScGT1 cell lines, to which Nbs themselves have not been shown to be neurotoxic (50, 74).

Concerning degenerative diseases of the central nervous system other than AD and PD, Nbs can also play a beneficial role in Huntington’s disease. Similar to AD and PD, Huntington’s disease is caused by a genetic mutation, in this case one that causes abnormal Huntingtin proteins to accumulate in nerve cells and affect nerve cell function. The basal ganglia is usually the first to be affected, causing the chorea symptoms. In 2015, the first Nb targeting the N-terminal domain of Huntingtin protein was developed and reported to show good affinity for human mutant and wild-type Huntingtin protein (135). The use of Nbs as an intrabody may be a good development direction given the intracellular aggregation of Huntington’s protein, and other sources of intrabody have been shown to be feasible early on (136).

There are also many diseases of the nervous system characterized by an inflammatory response, such as multiple sclerosis. Multiple sclerosis (MS) is a primary demyelinating disease of unknown etiology, which may be related to genetic, environmental, infection and other factors. The lesions are extensive, including significantly reduced oligodendrocyte and astrocyte proliferation. The current idea of treatment for MS is to reduce inflammation and promote myelin regeneration. Since multiple cytokines are involved in the mechanism of MS and there are no typical pathological molecules like in degenerative diseases, antibodies to treat MS usually target various cytokines and immune cell surface antigens. At present, the targets of Nbs that have been evaluated as a treatment option for MS are TNFR1 and CXCL10. The anti-TNFR1 Nb TROS was shown to inhibit inflammation *in vitro* and *in vivo (* 137), TROS also reduced neuroinflammation, preserved myelin and neurons in the MS model (mog35-55 induced experimental autoimmune encephalomyelitis, EAE) (138). The Nb 3Nb12 against CXCL10 was reported to block the CXCL10-CXCR3 binding and effectively inhibit the chemotaxis of CXCR3-Transfected HEK293T cells, which provides support for subsequent studies on treatment and diagnosis (139).

ALX-0681, with commercial name caplacizumab, is a Nb targeting the A1 domain of vWF, and is able to instantly inhibit the formation of microthrombi, significantly reducing the risk of thrombosis in acquired thrombocytopenic purpura (aTTP) (140). It was approved by the European Commission (EC) in 2018 for the treatment of adult patients with aTTP. Intriguingly, ALX-0681 was developed as an advanced variant of ALX-0081, also a bivalent vWF-targeting Nb. Before ALX-0681 went into clinical trials, ALX-0081 had been investigated in patients with risk to develop thromboembolism. Although it failed in a comparative Phase II randomized trial since it did not perform better in reducing bleeding events compared with abciximab (NCT01020383) (141), there was one study which demonstrated its capability in reducing brain infarction (142). Specifically, ALX-0081 was considered to block GPIb-vWF interaction, thus preventing the complete occlusion of cerebral blood vessels, promoting reperfusion, meanwhile mitigating the risk of bleeding. In a number of clinical trials of caplacizumab, stroke as a major event was used to evaluate the drug effect (143, 144), however, whether caplacizumab can be used as an antithrombotic drug in the context of cerebral infarction remains unclear. In view that the current drug treatment for thrombotic stroke comprising anti-platelets, anti-coagulation and thrombolysis, but this cannot avoid the risk of brain hemorrhage, we believe that the application of anti-vWF Nbs is worth exploring (**Supplementary Table 4**).

## 4 Discussion and outlook

As molecules with small molecular weight, high affinity and low immunogenicity, Nbs can be applied in a wide range of fields and also offer specific advantages as compared to ordinary full-size antibodies. For brain-related research, Nbs can be used to develop a variety of microscopy imaging tracers for specific targets, and Nbs can also serve as linkage molecules in genetic regulators of specific genes.

Nbs are not only tools to study brain disease-related molecules, but also can be developed into diagnostic reagents and therapeutic drugs. At present, although there is no lack of innovation in the use of Nbs for AD, PD, prion and other brain diseases, most compounds under development are still in an early research and preclinical phase. Studies of the therapeutic effect of the Nbs will need to go beyond counteracting toxicity to cultured nerve cells and address the effect on various pathological and neurological manifestations in the disease models. Thereby, successful crossing of the BBB remains an important attention point for diagnostic or therapeutic targeting in brain diseases. Yet, examples we have given such as that Nbs targeting the extracellular domain of the human insulin-like growth factor-1 receptor (IGF1R) coupled with the non-BBB crossing analgesic neuropeptide galanin also showed a dose-dependent analgesic effect, point to conditions where Nbs can indeed cross the BBB *in vivo* and exert a biological effect. Recent findings on autophagy dysregulation in AD, may offer an interesting possible new direction. It has been found that lysosomal acidification leads to a decrease in enzyme activity, which in turn reduces the ability to decompose Aβ (145). In addition, few research has been done on the treatment with Nbs related to microglia, a crucial cell in central nervous system diseases. Recent studies have shown that once activated by Aβ, microglia reach a chronic tolerant phase as a result of broad defects in energy metabolism and subsequently diminished immune responses, including cytokine secretion and phagocytosis. Interferon-gamma treatment reversed the defective glycolytic metabolism and inflammatory functions of microglia and thereby mitigated the AD pathology of 5XFAD mice (146). Microglia and their modulators may also be targeted with Nbs, either as tracers for imaging or even for therapeutics in case the suitable targets can be identified.

With regard to brain tumors, although Nbs and Nb-derived conjugates have many advantages for GB diagnosis and treatment, there are also some potential pitfalls. For instance, in case of systemic administration, effects on organs such as kidneys, liver or spleen may cause dose-limiting toxicities. In addition, chemical modification of Nbs may change their affinity, so it is necessary to verify the affinity and specificity of the Nbs before and after labeling or fusion with other compounds such as toxins. Moreover, the above literature provides valuable clues for the clinical application of Nbs, but before these schemes can be finally applied to the clinic many obstacles and difficulties still need to be overcome, the most important of which are the tumor heterogeneity of GB, and the individualized selection of the targets. Multi-target combination treatment strategies of immunotherapy and targeted therapy may be an important research direction to solve this problem in the future. Secondly, the evaluation of the treatment response of immunotherapy is also another problem. A robust imaging and molecular biology evaluation system will need to be established to predict and monitor the efficacy of therapy in individual patients, and there also Nbs may play a role. Also for the early detection of BMs, Nb-based molecular imaging holds great application prospects and, as a targeting molecule, Nb also greatly assists in drug delivery and radioimmunotherapy, allowing relatively mature treatments for peripheral lesions to have the opportunity to work in the brain. The ultimate requirement will mainly be clinical dose-response studies to address whether sufficient amounts of the Nbs can be obtained across the BBB to effectively eliminate cancer cells in the brain, under conditions with acceptable toxicity, both in the brain and in peripheral organs.

It is worth to mention that, in the current review, we have mainly focused on the use of Nbs as research tools and tracers for *in vivo* imaging and therapy. In addition, Nbs can also be used as tracers in microcantilever sensors (147) and organic transistors (148, 149) for early detection of diseases or pathogen detection. At the same time, the lower cost of producing Nbs compared to monoclonal antibodies makes them suitable for manufacturing *in vitro* diagnostic kits. At present, the research and development of high-sensitivity detection devices for the detection of brain diseases is still in a relatively early stage and warrants further studies. But biosensors using Nbs definitively show promising potential for rapid, low-cost and large-scale screening for brain diseases such as AD in the future (150).

Overall, there is still a lot of innovation to be explored in the application of Nbs in the field of brain diseases.

## Author contributions

FZ, YCP, LL, YXP, JZ, and XW wrote this manuscript. FZ and GR revised this manuscript. All authors contributed to the article and approved the submitted version.

## Funding

FZ was supported by National Natural Science Foundation of China(No. 38170187, Basic research program of Natural Science in Shaanxi Province (NO. 2021JM-007), Science Foundation of the Chinese Academy of Medical Sciences(2021-JKCS-008), LL, YCP, and XW were supported by national college students’ science and technology innovation project (SJ202110698176).

## Acknowledgments

We apologize for not including all of the publications by our colleagues.

## Conflict of interest

GR is shareholder of Precirix and Abscint and is inventor on various patent applications covering diagnostic and/or therapeutic use of Nbs. FZ is shareholder of Shaanxi Shaanxi Haisinuowei Technology Co. LTD and is inventor on various patent applications covering diagnostic and/or therapeutic use of Nbs.

The remaining authors declare that the research was conducted in the absence of any commercial or financial relationships that could be construed as a potential conflict of interest.

## Publisher’s note

All claims expressed in this article are solely those of the authors and do not necessarily represent those of their affiliated organizations, or those of the publisher, the editors and the reviewers. Any product that may be evaluated in this article, or claim that may be made by its manufacturer, is not guaranteed or endorsed by the publisher.
