# Supplementary material for: Applications of nanobodies in brain diseases
Source: Front Immunol. 2022 Nov 8;13:978513. doi: 10.3389/fimmu.2022.978513 (PMC9679430; doi:10.3389/fimmu.2022.978513)
Supplement: Supplementary file 1 [file Table_1.docx]

Supplementary Material

**Supplementary Table 1.** Nanobodies related to the diagnosis and treatment of AD

| Name | Epitope | K_D_ or k_cat_/K_m_ | structure | model | | BBB  Crossing | therapeutic  effects | Ref. |
| --- | --- | --- | --- | --- | --- | --- | --- | --- |
|  |  |  |  | *In vivo* | *In vitro/ex vivo* |  |  |  |
| R3VQ | Central Aβ | - | R3VQ-S-AF488 | PS2APP mice | brain sections of mice and human | yes | - | (1) |
|  |  |  | R3VQ-S-(DOTA/Gd)_3_ | PS2APP mice | mice brain sections |  |  | (2) |
| A2 | C terminal Tau | - | A2-S-AF488 | Tg4510 mice | brain sections of mice and human | yes | - | (1) |
| pa2H[76] | Aβ | 160 nM | pa2H-Alexa594  ^99m^Tc-pa2H | APP/PS1 mice | - | yes | - | (3) |
|  |  |  | pa2H-Fc | APP/PS1 mice | - | yes | - | (4) |
| VHH ni3A | Aβ | 39 nM | ni3A | - | BBB co-culture system | yes | - | (5) |
|  |  |  | ^99m^Tc-ni3A  ni3A-Alexa594 | APP/PS1 mice | - | yes | - | (3) |
| F8-2_R111C_[78] | C terminal Tau | 211 nM | F8-2_R111C_ | - | THY-Tau30 and KO-Tau transgenic mice brain sections | - | - | (6) |
| Nb_9 | mid-to carboxy-terminal domain of Aβ | 745±90 nM | Nb_9 | - | - | - | - | (7) |
| V31-1 | C terminal Aβ | 30 ±10 nM | V31-1 | - | mixing  SK-N-SH cells | - | inhibits the formation of fibril  prevents the Aβ-induced toxicity | (8) |
| B10 | Conformational epitope of Aβ | 475 ± 54 nM | B10AP | - | mixing | - | inhibits the formation of fibril | (9) |
|  |  |  | AGuIX@B10AP@Cy5.5 | - | APP/PS1 mice brain section | - | - | (10) |
| A4 | Aβ | - | A4 | - | mixing  SH-SY5Y cells | - | inhibits the formation of fibril  prevents the Aβ-induced toxicity | (11) |
| E1 | Aβ | - | E1 | - | mixing  SH-SY5Y cells | - | inhibits the formation of fibril  prevents the Aβ-induced toxicity | (12) |
| VHH Z70 | Tau PHF6 sequence | - | VHH Z70 | - | Biosensor reporter cell  mice brain section | - | inhibits Tau seeding | (13) |
| Asec-1A | α-secretase site | 58.07 μM/min | Asec-1A | - | mixing  SH-SY5Y Cells  7PA2 cell | - | hydrolyze Aβ  prevents the Aβ-induced toxicity | (14, 15) |

**Supplementary Table 2.** Nanobodies associated with PD diagnosis and treatment

| name | Epitope | K_D_ | structure | model | | therapeutic  effects | Ref. |
| --- | --- | --- | --- | --- | --- | --- | --- |
|  |  |  |  | *In vivo* | *In vitro/ex vivo* |  |  |
| NbSyn2 | C terminal α-synuclein | 100 nM | NbSyn2 | - | Mixing  Cell culture | inhibit the formation ofα-synuclein fibrils  reduced α-synuclein induced cellular damage | (16) |
| NbSyn87 | C terminal α-synuclein | 20 nM | NbSyn87 | - | Mixing  Cell culture | inhibit the formation ofα-synuclein fibrils  reduced α-synuclein induced cellular damage | (16) |
|  |  |  | NbSyn87PEST intracellular antibodies | α-synuclein overexpression rodent PD model | ST14A cells | α-synuclein degradation | (17, 18) |
| Nbα-syn01 | N terminal α-synuclein | 244 nM | Nbα-syn01  BivNb -syn01 | - | mixing  SH- SY5Y cells  Human brain tissue | Inhibit α-synuclein seeded fibril formation  Reduce α-synuclein seeded induced toxicity | (19) |
| Nb17 | Full length LRRK2 | 2.0±0.3 nM | Nb17 | - | HEK293 cells | inhibit LRRK2 kinase activity | (20) |

**Supplementary Table 3.** Nanobodies associated with the theragnostics of brain tumors

| Name | Target and domain properties | Function | Evidence | BBB crossing | Ref. |
| --- | --- | --- | --- | --- | --- |
| 7D12/9G8 | bivalent anti-EGFR Nb conjugated with TRAIL | Inhibit tumor proliferation and promote cell death;  sensitize tumor cells to TRAIL-induced apoptosis | *in vitro* Gli36, U87, and LN229 cells;  *in vivo*  U87-mCherry-FLuc xenografted mice | - | (21) |
| ENb | Anti-EGFR Nb conjugated with pseudomonas exotoxin (PE) | toxin-directed TRAIL sensitization of resistant tumor cells | *in vitro*  TRAIL-resistant LN229, U251, GBM23, GBM64 cells | - | (22) |
| US28-Nb^HLE^ | Bivalent anti-US28 Nb with an albumin-binding Nb | reduce angiogenesis and tumor growth | *in vivo* intraperitoneal administration to murine orthotopic xenograft models using human cell lines U251 *in vitro* U251 cells and GBM48 (primary GBM) cells both infected with HCMV Merlin strain | - | (23) |
| VUN100-PS | Anti-US28 Nb labelled with the photosensitizer, IRDye700DX | induce US28-expressing tumor cell death upon illumination with near-infrared light | *in vitro* US28 positive and negative U251 cells both in 2D and 3D cultures | - | (24) |
| NB225 | Anti-TUFM | inhibit GSCs growth with minor effects on astrocytes | *in vitro*  U251MG, U87MG, NCH644 and NCH421k cells, and astrocytes | - | (25) |
| NB79 | Anti-vimentin | inhibit migration of mature GSCs |  | - | (25) |
| NB237 | Anti-TRIM28 | inhibit tumor invasion | *in vitro*  U87, U373 cells  *in vivo*  zebra fish embryos injected with GB cells, U373 and U87, and NCH421k GSCs | - | (26) |
| EG_2_-hFc | Two anti-EGFR /EGFRvⅢ Nbs (EG_2_) fused with human Fc fragment and then labelled with Cy5.5 | target EGFR /EGFRvⅢ-expressing GB cells for optical imaging | *in vivo*  Mice intracerebrally implanted with U87MG.EGFRvⅢ tumor cells | Yes | (27) |
| sdAb 4.43 | A Gd-loaded vesicle labelled with Cy5.5 and conjugated with the anti-IGFBP7 Nb | target IGFBP7-expressing tumor vessels for optical and MRI bimodal imaging | *in vivo*  orthotopic U87MG.EGFRvIII glioblastoma xenografted mice | Yes | (28) |
| Nb15 | Monovalent and anti-SIRPα Nb labelled with ^99m^Tc | image SIRPα-expressing TAMs infiltrating the tumor microenvironment by SPECT | *in vivo*  mice bearing GL261 brain tumors, | Yes | (29) |
| Nb206 | Anti-mitochondrial translation elongation factor (TUFM) | inhibit GSCs growth | *in silico*  three-dimensional (3D) modelling of Nb206's binding pattern  *in vitro*  whole GSCs (NCH644 and NCH421K cells), U251MG, U87MG cells | - | (30) |
| 2Rs15d | Radiolabeled with [^18^F]RL-I | image HER2 expressing cells by PET  trastuzumab and/or pertuzumab therapy | *in vitro*  BT474M1 breast carcinoma cells  *in vivo*  in subcutaneously intracranial xenografts | yes | (31) |
|  | fluorescently labelled with quantum dots | detect micrometastases of HER2-positive tumors  using single- and two-photon imaging | *ex vivo*  biological samples of all major organs of HER2 positive breast tumor mouse models, which were transplanted with BT474 into the right inguinal mammary gland, or with SKBR3 cells to the liver | yes | (32) |
|  | labeled with ^111^In | for BM detection via μSPECT/CT | *ex vivo*  biological samples of all major organs of mice bearing either intracranial SKOV3.IP1 or 231Br tumor  *in vivo*  whole-body and brain-focused scan of the mice | yes | (33) |
|  | labeled with ^131^I using Sn-precursor of SGMIB | for HER2^pos^ lesion diagnosis and β^-^-radionuclide therapy; | *in vivo*  mice bearing trastuzumab-resistant 231Br tumor |  | (33) |
|  | labled with ^225^Ac using DOTA-based bifunctional chelators | for α- radionuclide therapy | *in vivo*  mice bearing small trastuzumab-sensitive SKOV3.IP1 | - | (33) |
| T12/P-Lipo | A dual-targeting liposomal SV/Gef-codelivery system  modified with an anti-PDL1 Nb and a T12 peptide | (promote TAM M2→M1 repolarization and anti-angiogenesis)  reverse EGFR^T790M^-associated drug resistance,  overcome brain metastasis of NSCLC | *in vitro* a transwell co-culture system of macrophages in the higher chamer and HUVEC (or H197 tumor cells) in the lower chamber  *in vivo* a BMs-bearing mouse model developed by intracranial transplanting the H1975 NSCLC cells | - | (34) |

**Supplementary Table 4.** Nanobodies associated with other CNS diseases

| name | Related diseases | structure | Model | | BBB crossing | therapeutic  effects | Ref. |
| --- | --- | --- | --- | --- | --- | --- | --- |
|  |  |  | *In vivo* | *In vitro/ex vivo* |  |  |  |
| VHH_G9_、VHH_F3_ | invasive meningococcal disease | VHH_G9_、VHH_F3_ | - | BBB model | - | reduce crossing of *neisseria meningitidis* in BBB Model | (35) |
| 26424, 26434 | Rabies disease | pentavalent multimers fused with COMP48 | Kunming mouse | BHK-21 cells | - | Neutralize the virus | (36) |
| rabe-e8 /H7 | Rabies disease | bivalent or biparatopic multimers through linkage with anti- albumin VHH | Swiss outbred mice | BHK-21 cells | - | Neutralize the virus | (37) |
| Nb484 | prion diseases | Nb484 | - | ScGT1 cell line  cerebellar organotypic cultured slices from tga20 transgenic | - | inhibit prion conversion | (38, 39) |
| PrioV3 antibody | prion diseases | PrioV3 antibody | healthy mice | in vitro BBB model  ScGT1 cell lines  RML-infected mice brain section | Yes | inhibit prion conversion | (40) |
|  |  | PrioV3 antibody | Rat | ScN2a Cells  GPNT rat and D3 human BBB models. | Yes | inhibits accumulation of PrP^Sc^ | (41) |
| TROS | multiple sclerosis | Trivalent bispecific nanobody targeting albumin and TNFR1 | EAE | HEK-2 blue cells  HEK293T Cells | - | Selective TNF/TNFR1 inhibition | (42, 43) |
| 3Nb12 | multiple sclerosis | 3Nb12 | - | HEK293T Cells | - | blocked CXCL10- CXCR3 binding | (44) |
| ALX-0081 | Aquired TTP and ischemic stroke | bivalent construct containing two identical VHH against vWF | - | brains of guinea pigs with middle cerebral artery (MCA) thrombosis | - | reduce thrombosis; induce reperfusion | (45) |

**References**

1. Li T, Vandesquille M, Koukouli F, Dudeffant C, Youssef I, Lenormand P, et al. Camelid Single-Domain Antibodies: A Versatile Tool for in Vivo Imaging of Extracellular and Intracellular Brain Targets. *J Control Release* (2016) 243:1-10.

2. Vandesquille M, Li T, Po C, Ganneau C, Lenormand P, Dudeffant C, et al. Chemically-Defined Camelid Antibody Bioconjugate for the Magnetic Resonance Imaging of Alzheimer's Disease. *MAbs* (2017) 9(6):1016-27.

3. Nabuurs RJ, Rutgers KS, Welling MM, Metaxas A, de Backer ME, Rotman M, et al. In Vivo Detection of Amyloid-Beta Deposits Using Heavy Chain Antibody Fragments in a Transgenic Mouse Model for Alzheimer's Disease. *PLoS One* (2012) 7(6):e38284.

4. Rotman M, Welling MM, van den Boogaard ML, Moursel LG, van der Graaf LM, van Buchem MA, et al. Fusion of Higg1-Fc to 111in-Anti-Amyloid Single Domain Antibody Fragment Vhh-Pa2h Prolongs Blood Residential Time in App/Ps1 Mice but Does Not Increase Brain Uptake. *Nucl Med Biol* (2015) 42(8):695-702.

5. Rutgers KS, Nabuurs RJ, van den Berg SA, Schenk GJ, Rotman M, Verrips CT, et al. Transmigration of Beta Amyloid Specific Heavy Chain Antibody Fragments across the in Vitro Blood-Brain Barrier. *Neuroscience* (2011) 190:37-42.

6. Dupre E, Danis C, Arrial A, Hanoulle X, Homa M, Cantrelle FX, et al. Single Domain Antibody Fragments as New Tools for the Detection of Neuronal Tau Protein in Cells and in Mice Studies. *ACS Chem Neurosci* (2019) 10(9):3997-4006.

7. Paraschiv G, Vincke C, Czaplewska P, Manea M, Muyldermans S, Przybylski M. Epitope Structure and Binding Affinity of Single Chain Llama Anti-Beta-Amyloid Antibodies Revealed by Proteolytic Excision Affinity-Mass Spectrometry. *J Mol Recognit* (2013) 26(1):1-9.

8. Lafaye P, Achour I, England P, Duyckaerts C, Rougeon F. Single-Domain Antibodies Recognize Selectively Small Oligomeric Forms of Amyloid Beta, Prevent Abeta-Induced Neurotoxicity and Inhibit Fibril Formation. *Mol Immunol* (2009) 46(4):695-704.

9. Habicht G, Haupt C, Friedrich RP, Hortschansky P, Sachse C, Meinhardt J, et al. Directed Selection of a Conformational Antibody Domain That Prevents Mature Amyloid Fibril Formation by Stabilizing Abeta Protofibrils. *Proc Natl Acad Sci U S A* (2007) 104(49):19232-7.

10. Pansieri J, Plissonneau M, Stransky-Heilkron N, Dumoulin M, Heinrich-Balard L, Rivory P, et al. Multimodal Imaging Gd-Nanoparticles Functionalized with Pittsburgh Compound B or a Nanobody for Amyloid Plaques Targeting. *Nanomedicine (Lond)* (2017) 12(14):1675-87.

11. Zameer A, Kasturirangan S, Emadi S, Nimmagadda SV, Sierks MR. Anti-Oligomeric Abeta Single-Chain Variable Domain Antibody Blocks Abeta-Induced Toxicity against Human Neuroblastoma Cells. *J Mol Biol* (2008) 384(4):917-28.

12. Kasturirangan S, Li L, Emadi S, Boddapati S, Schulz P, Sierks MR. Nanobody Specific for Oligomeric Beta-Amyloid Stabilizes Nontoxic Form. *Neurobiol Aging* (2012) 33(7):1320-8.

13. Danis C, Dupre E, Zejneli O, Caillierez R, Arrial A, Begard S, et al. Inhibition of Tau Seeding by Targeting Tau Nucleation Core within Neurons with a Single Domain Antibody Fragment. *Mol Ther* (2022) 30(4):1484-99.

14. Kasturirangan S, Brune D, Sierks M. Promoting Alpha-Secretase Cleavage of Beta-Amyloid with Engineered Proteolytic Antibody Fragments. *Biotechnol Prog* (2009) 25(4):1054-63.

15. Kasturirangan S, Boddapati S, Sierks MR. Engineered Proteolytic Nanobodies Reduce Abeta Burden and Ameliorate Abeta-Induced Cytotoxicity. *Biochemistry* (2010) 49(21):4501-8.

16. El-Turk F, Newby FN, De Genst E, Guilliams T, Sprules T, Mittermaier A, et al. Structural Effects of Two Camelid Nanobodies Directed to Distinct C-Terminal Epitopes on Alpha-Synuclein. *Biochemistry* (2016) 55(22):3116-22.

17. Butler DC, Joshi SN, Genst E, Baghel AS, Dobson CM, Messer A. Bifunctional Anti-Non-Amyloid Component Alpha-Synuclein Nanobodies Are Protective in Situ. *PLoS One* (2016) 11(11):e0165964.

18. Chatterjee D, Bhatt M, Butler D, De Genst E, Dobson CM, Messer A, et al. Proteasome-Targeted Nanobodies Alleviate Pathology and Functional Decline in an Alpha-Synuclein-Based Parkinson's Disease Model. *NPJ Parkinsons Dis* (2018) 4:25.

19. Hmila I, Vaikath NN, Majbour NK, Erskine D, Sudhakaran IP, Gupta V, et al. Novel Engineered Nanobodies Specific for N-Terminal Region of Alpha-Synuclein Recognize Lewy-Body Pathology and Inhibit in-Vitro Seeded Aggregation and Toxicity. *FEBS J* (2022).

20. Leemans M, Galicia C, Deyaert E, Daems E, Krause L, Paesmans J, et al. Allosteric Modulation of the Gtpase Activity of a Bacterial Lrrk2 Homolog by Conformation-Specific Nanobodies. *Biochem J* (2020) 477(7):1203-18.

21. Van De Water JAJM, Bagci-Onder T, Agarwal AS, Wakimoto H, Roovers RC, Zhu Y, et al. Therapeutic Stem Cells Expressing Variants of Egfr-Specific Nanobodies Have Antitumor Effects. *Proceedings of the National Academy of Sciences of the United States of America* (2012) 109(41):16642-7. doi: 10.1073/pnas.1202832109.

22. Karakaş N, Stuckey D, Revai-Lechtich E, Shah K. *Il13rα2- and Egfr-Targeted Pseudomonas Exotoxin Potentiates the Trail-Mediated Death of Gbm Cells*. International Journal of Molecular Medicine(2021).doi: 10.3892/IJMM.2021.4978.

23. Heukers R, Fan TS, de Wit RH, van Senten JR, De Groof TWM, Bebelman MP, et al. The Constitutive Activity of the Virally Encoded Chemokine Receptor Us28 Accelerates Glioblastoma Growth. *Oncogene* (2018) 37(30):4110-21.

24. De Groof TWM, Mashayekhi V, Fan TS, Bergkamp ND, Toraño JS, Van Senten JR, et al. Nanobody-Targeted Photodynamic Therapy Selectively Kills Viral Gpcr-Expressing Glioblastoma Cells. *Molecular Pharmaceutics* (2019). doi: 10.1021/acs.molpharmaceut.9b00360.

25. Zottel A, Jovčevska I, Šamec N, Mlakar J, Šribar J, Križaj I, et al. Anti-Vimentin, Anti-Tufm, Anti-Nap1l1 and Anti-Dpysl2 Nanobodies Display Cytotoxic Effect and Reduce Glioblastoma Cell Migration. *Therapeutic Advances in Medical Oncology* (2020) 12. doi: 10.1177/1758835920915302.

26. Porčnik A, Novak M, Breznik B, Majc B, Hrastar B, Šamec N, et al. Trim28 Selective Nanobody Reduces Glioblastoma Stem Cell Invasion. *Molecules* (2021) 26(17). doi: 10.3390/molecules26175141.

27. Iqbal U, , Trojahn U, , Albaghdadi H, , et al. Kinetic Analysis of Novel Mono- and Multivalent Vhh-Fragments and Their Application for Molecular Imaging of Brain Tumours: Research Paper. (2010) 160:1016-28. doi: 10.1111/j.1476-5381.2010.00742.x.

28. Iqbal U, , Albaghdadi H, , Luo Y, , et al. Molecular Imaging of Glioblastoma Multiforme Using Anti-Insulin-Like Growth Factor-Binding Protein-7 Single-Domain Antibodies. (2010) 103:1606-16. doi: 10.1038/sj.bjc.6605937.

29. De Vlaminck K, , Romão E, , Puttemans J, , et al. Imaging of Glioblastoma Tumor-Associated Myeloid Cells Using Nanobodies Targeting Signal Regulatory Protein Alpha. (2021) 12:1-14. doi: 10.3389/fimmu.2021.777524.

30. Samec N, Jovcevska I, Stojan J, Zottel A, Liovic M, Myers MP, et al. Glioblastoma-Specific Anti-Tufm Nanobody for in-Vitro Immunoimaging and Cancer Stem Cell Targeting. *Oncotarget* (2018) 9(25):17282-99.

31. Zhou Z, Vaidyanathan G, McDougald D, Kang CM, Balyasnikova I, Devoogdt N, et al. Fluorine-18 Labeling of the Her2-Targeting Single-Domain Antibody 2rs15d Using a Residualizing Label and Preclinical Evaluation. *Molecular Imaging and Biology* (2017) 19(6):867-77. doi: 10.1007/s11307-017-1082-x.

32. Ramos-Gomes F, Bode J, Sukhanova A, Bozrova SV, Saccomano M, Mitkovski M, et al. Single- and Two-Photon Imaging of Human Micrometastases and Disseminated Tumour Cells with Conjugates of Nanobodies and Quantum Dots. *Scientific Reports* (2018) 8(1):1-12. doi: 10.1038/s41598-018-22973-8.

33. Puttemans J, Dekempeneer Y, Eersels JL, Hanssens H, Debie P, Keyaerts M, et al. Preclinical Targeted Α- and Β −-Radionuclide Therapy in Her2-Positive Brain Metastasis Using Camelid Single-Domain Antibodies. *Cancers* (2020) 12(4). doi: 10.3390/cancers12041017.

34. Yin W, Zhao Y, Kang X, Zhao P, Fu X, Mo X, et al. Bbb-Penetrating Codelivery Liposomes Treat Brain Metastasis of Non-Small Cell Lung Cancer with Egfrt790m Mutation. *Theranostics* (2020) 10(14):6122-35. doi: 10.7150/thno.42234.

35. Kulkarni A, Mochnacova E, Majerova P, Curlik J, Bhide K, Mertinkova P, et al. Single Domain Antibodies Targeting Receptor Binding Pockets of Nada Restrain Adhesion of Neisseria Meningitidis to Human Brain Microvascular Endothelial Cells. *Front Mol Biosci* (2020) 7:573281.

36. Boruah BM, Liu D, Ye D, Gu TJ, Jiang CL, Qu M, et al. Single Domain Antibody Multimers Confer Protection against Rabies Infection. *PLoS One* (2013) 8(8):e71383.

37. Terryn S, Francart A, Lamoral S, Hultberg A, Rommelaere H, Wittelsberger A, et al. Protective Effect of Different Anti-Rabies Virus Vhh Constructs against Rabies Disease in Mice. *PLoS One* (2014) 9(10):e109367.

38. Abskharon RN, Giachin G, Wohlkonig A, Soror SH, Pardon E, Legname G, et al. Probing the N-Terminal Beta-Sheet Conversion in the Crystal Structure of the Human Prion Protein Bound to a Nanobody. *J Am Chem Soc* (2014) 136(3):937-44.

39. Abskharon R, Wang F, Wohlkonig A, Ruan J, Soror S, Giachin G, et al. Structural Evidence for the Critical Role of the Prion Protein Hydrophobic Region in Forming an Infectious Prion. *PLoS Pathog* (2019) 15(12):e1008139.

40. David MA, Jones DR, Tayebi M. Potential Candidate Camelid Antibodies for the Treatment of Protein-Misfolding Diseases. *J Neuroimmunol* (2014) 272(1-2):76-85.

41. Jones DR, Taylor WA, Bate C, David M, Tayebi M. A Camelid Anti-Prp Antibody Abrogates Prp Replication in Prion-Permissive Neuroblastoma Cell Lines. *PLoS One* (2010) 5(3):e9804.

42. Steeland S, Puimege L, Vandenbroucke RE, Van Hauwermeiren F, Haustraete J, Devoogdt N, et al. Generation and Characterization of Small Single Domain Antibodies Inhibiting Human Tumor Necrosis Factor Receptor 1. *J Biol Chem* (2015) 290(7):4022-37.

43. Steeland S, Van Ryckeghem S, Van Imschoot G, De Rycke R, Toussaint W, Vanhoutte L, et al. Tnfr1 Inhibition with a Nanobody Protects against Eae Development in Mice. *Sci Rep* (2017) 7(1):13646.

44. Sadeghian-Rizi T, Behdani M, Khanahmad H, Sadeghi HM, Jahanian-Najafabadi A. Generation and Characterization of a Functional Nanobody against Inflammatory Chemokine Cxcl10, as a Novel Strategy for the Treatment of Multiple Sclerosis. *CNS Neurol Disord Drug Targets* (2019) 18(2):141-8.

45. Momi S, , Tantucci M, , Van Roy M, , et al. Reperfusion of Cerebral Artery Thrombosis by the Gpib-Vwf Blockade with the Nanobody Alx-0081 Reduces Brain Infarct Size in Guinea Pigs. (2013) 121:5088-97. doi: 10.1182/blood-2012-11-464545.
